# Patients' Experience on Practice and Applicability of Informed Consent in Traditional Medical Practice in KwaZulu-Natal Province, South Africa

**DOI:** 10.1155/2022/3674467

**Published:** 2022-01-20

**Authors:** Francis Akpa-Inyang, Elizabeth Ojewole, Sylvester C. Chima

**Affiliations:** ^1^Programme of Bio & Research Ethics and Medical Law, School of Nursing and Public Health, College of Health Sciences, University of KwaZulu-Natal, Durban, South Africa; ^2^Discipline of Pharmaceutical Sciences, School of Health Sciences, University of KwaZulu-Natal (Westville Campus), P.O. Box X54001, Durban 4000, South Africa; ^3^Programme of Bio & Research Ethics and Medical Law, School of Nursing and Public Health, and Nelson R Mandela School of Medicine, College of Health Sciences, University of KwaZulu-Natal, Durban, South Africa

## Abstract

**Background:**

Informed consent (IC) is constitutionally protected in South Africa based on individual rights to bodily integrity and well-being. In terms of the law, patients cannot be involved in medical treatment or research without IC. This study explored patients' experience on practice and applicability of IC in African traditional medicine (ATM) in Msunduzi and eThekwini municipalities, KwaZulu-Natal province, South Africa, to evaluate whether important elements of IC such as full information disclosure, capacity, understanding, and volition are considered or being applied during ATM.

**Methods:**

This cross-sectional quantitative study was conducted using semistructured questionnaires administered to patients attending traditional health practitioners' (THPs') treatment centres. Stata V15.1 was used to analyse variables including descriptive and inferential data analysis.

**Results:**

One hundred and twenty-nine (129) participants completed this study, of which 62% were females. Most participants were in the age range of 26–35 (38.8%). All respondents were IsiZulu home-language speakers, single (62.8%), employed (48%), and with some tertiary education (48.8%). Most patients were informed about their diagnosis (58.9%), treatment benefits (79.8%), and recommended treatment (79.8%). Fewer were informed about risks of treatment (36.4%), right of refusal (3.1%), and risks of refusing recommended treatment (0.8%). All participants reported satisfaction with information disclosed by the THPs and did not feel coerced to accept treatment. Consent was obtained verbally in all cases. The majority of participants (76.7%) sought surrogate assistance when consulting THPS, and 81.4% preferred being informed about all treatment risks. Most respondents also preferred involvement in healthcare decision-making during ATM.

**Conclusion:**

This study reveals that most patients consulting THPs in the KwaZulu-Natal province for treatment are aware of their right to information disclosure and the need to reach agreement before involvement in ATM treatment procedures. The study also showed that some key elements of IC are currently being applied during ATM practice in South Africa.

## 1. Introduction

It is widely believed that knowledge gives freedom. For one to choose the most appropriate course of action, he/she must appreciate all aspects of a situation and all the possible alternatives. We need information to make the right decisions, to manage our behaviours, and to be independent. Being adequately informed before decision-making can therefore be considered a fundamental human right since appropriate behaviour depends on information [1]. Informed consent (IC) during treatment is recognized as both an ethical and a legal principle, which is founded on the principle of respect for individual autonomy and a patient's “right” to self-determination [2–4].

The principle of IC is an ethical and legal principle, which stipulates that patients must not be involved in any diagnostic or therapeutic procedure unless the patient is informed of the risks of the procedure and its alternatives prior to giving consent [4–7]. Informed consent has been defined as the autonomous approval of medical intervention by an individual patient [4–7]. As a general rule, healthcare treatment should not be given unless the healthcare professional has previously obtained the patient's explicit or implicit consent when the patient presents to a medical doctor or any other healthcare professional for consultation before following the recommended routine [8–10].

In this paper, African traditional medicine (ATM) refers to a diverse field of traditional medicine as practiced in South Africa, which would include traditional healing interwoven with cultural practices and spiritual beliefs that are regarded as being holistic, involving both the body and the mind [11–14]. African traditional medicine has an important role to play in healthcare delivery among African societies [15–17]. Traditional healthcare practitioners (THPs) are usually the first port of call before consultations with Western orthodox or biomedical practitioners (BMPs) and the last resort when biomedical intervention efforts fail [16–18]. It is estimated that there are between 250 000 and 400 000 traditional healers in South Africa compared with 28 000 medical doctors [16]. Furthermore, eight out of every 10 black Africans may rely on traditional medicine alone, or in combination with Western medicine [16–18]. This is because THPs occupy esteemed positions within many indigenous African cultures as they are consulted for a wide range of physical, social, and emotional problems and often assumed multiple roles as a medicinal healer, physician, priest, psychiatrist, advisor, teacher, diviner, and herbalist in their communities [12–18].

South African THPs may register as members of the interim Traditional Health Professions Council of South Africa (THPCSA) in accordance with government regulations published in 2015 [19], consistent with the Traditional Health Practitioners Act of 2007 [20]. Hence, the THPCSA may require that like other healthcare professionals registered with health professions councils in South Africa, such as the Health Professions Council of South Africa (HPCSA) [4, 6, 9], and the South African Nursing Council (SANC) [21], consistent with the National Health Act 2003 (NHA) [22]. Ultimately, THPs may also be required to obtain valid IC from their patients prior to treatment, in accordance with current law [4, 6, 9, 22, 23].

However, some commentators have suggested that the biomedical model of IC is not applicable in ATM because there are two forms of traditional medicine: the supernatural and herbalism [24]. Whereas the supernatural represents a process whereby ancestral spirits diagnose the cause of illness and reveal the right treatment, herbalism is the use of herbs for therapeutic purposes [24–26]. Traditional medicine as practiced in South Africa is of different types, that is why there are many THP roles, which includes herbalists (*izinyanga* or *amaxhwele*), diviners (*izangoma*, *umthandazi*, or *amagqirha*), traditional surgeons (*iingcibi*) who mainly do circumcision, and traditional birth attendants (*ababelethisi* or *abazalisi*) [12–14, 18, 27–29]. This diversity also implies the application of diverse diagnostic tools and different treatment modalities [12–14, 18, 24, 27–34]. Previous research from South Africa has shown that IC is applicable in the context of African traditional values and belief systems if the emphasis is place on care, honesty, respect, and fairness instead of an emphasis on rugged libertarian rights-based individualism [26, 30, 35]. This study explored patients' experience on practice and applicability of IC in ATM within Msunduzi and eThekwini municipalities, South Africa, to evaluate whether the key elements of IC such as full information disclosure, capacity, understanding, and voluntariness (absence of coercion or undue pressure) are considered or applied during ATM practice. This is to relate it with prevailing ethical values and the regulatory requirements for IC in South Africa and also to assess the possibility and extent of applying IC to ATM practice.

## 2. Methods

This study was a quantitative, descriptive, cross-sectional study in contemporary ATM practice settings in the Kwazulu-Natal province (KZN), South Africa. The cross-sectional design is suggested as the most relevant design when one is testing the knowledge, attitude, and perspective of patients about a phenomenon [36] due to the fact that this design is observational and also applicable to descriptive research studies [36]. This study design allowed the researchers to explore the experience of patients on the practice and applicability of IC in ATM practice in South Africa and was also cost-effective, which enabled data to be collected over a short space of time without the need to follow up with patients [36].

The study was conducted in the Msunduzi municipality (Pietermaritzburg) and eThekwini metropolitan municipality (Durban), KZN, South Africa. The KwaZulu-Natal province was created in 1994 when the Zulu Bantustan of KwaZulu and Natal Province were merged. It is located in the southeast of the country enjoying a long shoreline beside the Indian Ocean. Its capital is Pietermaritzburg, and the largest urban city is Durban. It is the second most populous province in South Africa with a population of 10,267,300 [37]. The major population groups within KZN are black Africans (86.8%), Indians/Asians (7.4%), whites or Caucasians (4.2%), Coloureds (1.4%) [26], and others including migrant Africans and citizens from other countries resident in South Africa (0.3%) [37]. The predominant languages spoken in KZN are IsiZulu (77.8%), English (13.2%), and IsiXhosa (3.4%) [4, 6, 9, 23, 37, 38]. This is a province with a lot of black African peoples within urban and rural communities where a research study on African traditional medicine could be done effectively.

### 2.1. Participants

This study targeted patients or individuals who had availed the services of a THP for treatment in the areas of herbalism, bone-setting, and midwifery within the past 5 years. The sample was limited to the past 5 years for authentication of the information reported. This is because the older the experience, the possibility of the patients forgetting the details of their experience or interactions with the THP is high.

Hence, patients who have been to THPs for herbal treatment, bone-setting, or maternal healthcare services within the last 5 years and who were willing to participate voluntarily were included in the study; whilst patients who have not been to THPs or had not used any of the proposed healthcare services were excluded. In addition, patients who were mentally incapacitated or below the age of consent for routine treatment as stipulated by local laws or who were unwilling to participate voluntarily were also excluded from the study.

The sample size for this study was determined using a sample size calculator on Stata V15.1 [39] at a confidence interval of 95% with a width of confidence of 5% and the assumption that 20% of respondents may have received IC since previous studies had suggested that IC is not applicable in ATM practice [24]. The estimated sample size for a two-sample proportions test was based on Pearson's chi-squared test:

- Ho: p2 = p1 versus Ha: p2 ! = p1
- Study parameters:
- alpha = 0.0500
- power = 0.8000
- delta = 0.1500 (difference)
- p1 = 0.2000
- p2 = 0.3500

Thus, the estimated sample size for this study was 138.

In this study, we adopted convenience sampling in the recruitment process. This is because the first author (PI) with the assistance of the senior author/project supervisor was introduced to THPs, including *Sangomas* (*Izangoma:* pl) and *Nyanga* (*Izinyanga:* pl) who were members of the KZN traditional healers' association. These traditional healers shared their consultation times with the PI, thereby giving the PI a platform to visit the *isigodlo* (*Esigodlweni:* pl)—consulting and treatment facilities for the traditional healers (THPs)—to speak with the patients about the research study. Patients who were interested in participating in the research were thus recruited for inclusion in the study. The PI was assisted by a trained bilingual research assistant, who served as an IsiZulu-English language interpreter, when visiting different *Izangoma* and *Izinyanga* healing centres. These healing centres are also called *isigodlo (Esigodlweni)* by South African healers. This location is a hut where the *amadlozi* and *izithunywa* (ancestral spirits) reside and also serves as a consultation room for traditional healers where patients sit during consultations. Here, the PI with assistance from the research assistant distributed the IC documents and questionnaires and also interviewed the patients.

### 2.2. Data Collection

This cross-sectional quantitative study was conducted using semistructured questionnaires, which were originally formulated in English. The questionnaires were then translated into IsiZulu, the dominant language spoken by about 81% of the population in KZN [4, 6, 9, 23, 38]. Participants were interviewed by a trained bilingual research assistant and the principal investigator (PI). Those who preferred were allowed to complete the questionnaires by themselves. Participants had the option of completing questionnaires either in English or IsiZulu, depending on their personal preference.

The questionnaire used in this study was adapted with few amendments of questions from a validated questionnaire for patients previously used by Chima [6]. This questionnaire comprised three sections: section A was designed to collect the demographic characteristics of participants, section B asked questions on consent to treatment, and section C was designed to collect generic questions on IC from respondents [6] (see Supplementary Materials 1).

A pilot study was conducted among researchers and other knowledgeable healthcare workers who have been to traditional healers as patients or were working in the traditional medicine laboratory in the University of KwaZulu-Natal to ensure that the questionnaire addressed the questions to be answered by the study population. Content and face validity of the questionnaire were evaluated, with minor changes made to language and phrasing. Minor corrections were made on the translated copy of the questionnaire based on comments and responses obtained from the pilot study. Data collection for the study took place in rural communities and townships around Pietermaritzburg and Durban KZN [9, 37, 38] from September to October 2020 at the homes/consultation centres of THPs.

### 2.3. Data Analysis

Participant responses were allocated codes, which were then captured for statistical analysis. Descriptive statistics were used to summarize the data. Categories with small frequencies were combined to create larger categories suitable for statistical analysis. For instance, most of the participants were single and few participants were married, divorced, or separated; thus, these categories were combined to create a larger category. In the analysis of treatment information provided to patients, an overall score was created by counting the number of “yes” responses to the seven questions on informed consent. The scores were then dichotomized at the median: low (1–3) and high (4–7). The seven questions covered information on diagnosis, treatment options, risks of treatment, benefits of treatment, recommended treatment, and information on patients' right of refusal as stipulated by the National Health Act 2003 [22], and other current South African regulations [4, 6, 9, 23]. This was to assess where patients were provided information on less than 3 of the above areas; it was then considered that the information the person received was inadequate. Conversely, patients who received information in more than 3 of the above areas were reported as receiving adequate information.

Social and demographic characteristics associated with the scores were analysed using chi-squared or Fisher's exact tests. These statistical tests were also used to identify associations between the independent variables of age, gender, marital status, level of education, and occupation, against the seven questions on informed consent. This was to determine if these variables or demographic characteristics are associated with the amount of information disclosed to patients. A *p* value of <0.05 was considered statistically significant. Stata IC V15.1 was used in the statistical analysis [39]. Further analysis was done using logistic regression to confirm the results from the chi-squared tests.

The statistical parameters for this study including design and analysis of data were evaluated, conducted, and approved with the assistance of a qualified biostatistician from the College of Health Sciences (CHS), University of KwaZulu-Natal, South Africa.

### 2.4. Ethical Considerations

This study was reviewed and approved by the University of KwaZulu-Natal Biomedical Research and Ethics Committee (Reference No.: BREC/00000525/2019) and was also reviewed and approved by the KZN Department of Health, Knowledge, and Management Research Ethics Committee (Reference No.: KZ_201911_018). Permissions were also obtained from eThekwini District Health Department, and community heads approvals were also obtained for the communities visited. We also obtained permission from the THPs (*Izangoma* and *Izinyanga*) before visiting their consultation centres. IC was obtained from all participants after full information disclosure. In addition, consent forms were separated from participant responses and the study was coded and de-identified and is reported anonymously to protect patient's privacy and confidentiality.

## 3. Results

### 3.1. Sociodemographic Characteristics

One hundred and twenty-nine valid questionnaires were completed by respondents with one having some incomplete or missing data. Thus, the response rate for this study was 93% of the original estimated sample size of 138 participants. The majority of participants (98.4%, *n* = 127) completed the questionnaires by themselves, while two questionnaires were completed by parents/guardians who have been to a THP with their wards or children (1.6%, *n* = 2). Most participants were female (62%, *n* = 80) and single (62.8%, *n* = 81), while 29.5% (*n* = 38) were married participants. The mean age of study participants was 37 years. Most respondents were within the age groups of 26–35 years (38.8%, *n* = 50) and 36–45 years (29.5%, *n* = 38). All the participants were IsiZulu home-language speakers, while a few spoke both IsiZulu and English languages (8.5%, *n* = 11). Sixty-two respondents had secondary education (48.1%), 63 had some tertiary education (48.8%), and four reported completing primary education (3.1%, *n* = 4). Most participants reported being employed in formal sectors of the economy (48.1%, *n* = 62), followed by the unemployed (36.4%, *n* = 47), while others were self-employed (15.5%, *n* = 20). Most participants (40.3%, *n* = 52) declined to reveal their income or earnings. Regarding those that revealed earnings, thirty-eight were unemployed and had no earning (29.5%), while 39 (30.2%) reported a monthly income. The demographic characteristics of the participants are summarised in Table 1.

### 3.2. Information Disclosure

This part of the study explores IC practice among THPs patronized by respondents. All the respondents in the study had been to a traditional healer within the past 5 years. Most of the respondents visited a *Sangoma* (57% *n* = 74); 54 (42%) visited an *Nyanga*, while only one (0.8%, *n* = 1) reported visiting a traditional orthopaedic healer (bonesetter).

All the participants (*n* = 129; 100%) reported that the traditional healer (THP) explained the treatment or procedure to them. Most participants were informed of their diagnosis (58.9%, *n* = 76), benefits of treatment (79.8%, *n* = 103), and recommended treatment (79.8%, *n* = 103). Few participants were informed about treatment options (14.7%, *n* = 19), while less than half of the participants were informed about the risks of treatment (36.4%, *n* = 47), and less than 3% of the participants were given information on their right of refusal (3.1%, *n* = 4), and risks of refusing recommended treatment (0.8%, *n* = 1).

Information disclosure had no relationship with the age (*ρ* = 0.54) or gender (*ρ* = 0.33) of respondents. However, there was an association with marital status (*ρ* = 0.004). That is, *married* patients were more likely to receive more information on treatment when compared with *single* patients. The level of education was also associated with more information disclosure (*ρ* = 0.005), that is, patients with secondary and tertiary education were more likely to get more information disclosed to them when compared with patients with primary education. Furthermore, occupational status had an association with information disclosure to patients (*ρ* = 0.02), in that employed patients were more likely to get more information disclosed to them, when compared with patients who were unemployed or self-employed (Tables 2 and 3).

Further analysis on these associations was performed using logistic regression. On bivariate analysis, marital status was significantly associated with informed consent. Thus, married/divorced/separated respondents were more than three times as likely to get more information on treatment procedures when compared with single respondents (OR: 3.6, 95% CI: 1.45–9.10). Employment status was also significantly associated with IC, whereby employed respondents are almost 3 times as likely to get information on treatment procedures when compared with unemployed respondents (OR: 2.8, 95%CI: 1.2–6.92). Self-employed respondents did not differ from the unemployed. In multivariate tests, all variables were included, although sometimes we only included variables with a *p* value of <0.3 to avoid overfitting the model. Results from multivariate logistic regression analysis confirmed that marital status remained significantly associated with IC after adjusting for possible confounders and with a similar OR. On the other hand, occupation was no longer significant after adjusting for other possible confounders, but the *p*-value could be considered borderline. The fact that the OR is still >2 indicates that it could be an important factor. Results of the logistic regression analysis are summarised in Table 4.

Furthermore, most participants (91.5%, *n* = 118) said “yes” when asked if the THPs advised them whether they could accept or reject the treatment procedure. Eight (6.2%) participants said “no,” while 3 (2.3%) said they “do not know.” When asked if they felt threatened, or afraid to say “no” to proposed treatment, 6 (4.7%) answered “yes” and 122 (94.6%) said “no,” while one participant said they “do not know.” The few participants that answered “yes” said it was because there is a general idea that traditional healers (THPs) know everything they are doing because South African traditional healers represent the ancestors. Therefore, one cannot say “no” to any proposed treatment. All participants answered “no” when they were asked if they were given any perverse incentive or persuaded to accept any particular treatment.

All respondents also said they understood the information provided. When asked if they asked any question about the treatment, 126 (97.7%) participants said “yes,” while 3 (2.3%) said “no.” The reason for the few that said “no” is that they trust the traditional healer and that the healers know what they are doing.

### 3.3. Decision-Making and Consenting

All respondents reported that consent/agreement was obtained verbally and information disclosure to patients was rendered using words in all cases. IsiZulu language was used in all the communication processes between the THPs and their patients. There was no other method used to enhance information disclosure.

Furthermore, all respondents reported that the information provided by the traditional healer (THP) was enough. When asked whether they made the treatment decision of their own free will, all the participants said “yes.” However, 40.0% (*n* = 52) of the participants went further to render open-ended comments on the questionnaire that coming to the THP means you are ready to do what he/she tells you to do. Thus, it is in rare cases that you see a patient refusing or questioning the traditional healers' prescriptions. When asked if they could change their mind or refuse treatment from the THP if they did not like the treatment, 127 (98.4%) respondents answered “yes,” and only one respondent (0.8%) said “no.” The other remaining respondent answered “do not know.”

When participants were asked if they sought assistance from any other person before deciding to accept or reject the recommended treatment, most participants (76.7%, *n* = 99) said “yes,” while 30 (23.3%) said “no.” Among those who sought assistance from surrogates, 29 (22.5%) were from parents, 4 (3.1%) sought help from husbands, 11 (8.5%) involved their wives, 33 (25.6%) sought help from other family members, and 22 (17.1%) involved a friend. The participants who sought assistance said they did so because they wanted some support. Others wanted to be sure they are doing the right thing, and some were consulting a THP for the very first time, so they needed someone who has been there before to guide them through the process. There was no significant association between occupation (*p* = 0.59) and level of education (*p* = 0.15) with seeking assistance. However, there was a significant association between being single and seeking assistance (*ρ* = 0.04). Also, male patients were more likely to seek assistance although this was of borderline significance (*ρ* = 0.059). In addition, all persons in the youngest age group (18–25) sought assistance (*ρ* = 0.02).

The study revealed that time spent by patients with traditional healers (THPs) during therapeutic encounter ranged from 5 to 10 minutes (31%, *n* = 40), 10 to 20 minutes (51.2%, *n* = 66), and 20 to 30 minutes (17.8%, *n* = 23) as reported by respondents.

### 3.4. Patients' Choice on Risk Disclosure

Most participants (81.4%, *n* = 105) reported that they would prefer to receive information on “all the risks” associated with the treatment or procedure. One participant (0.8%) said they would like to know only “some of the risk.” Some participants (16.3%, *n* = 21) said they would like to know “none of the risks,” while 2 (1.6%) participants said they “do not know.” Some reasons why many participants wanted full disclosure of the risk include that they wanted to know what treatment/medications they are taking, which would help them to decide if they want it or not. Some also wanted to know the side effects of the medications. Most of those who preferred partial or nondisclosure of all risks said it is because they trusted the traditional healer. When association was tested with age, there was a strange pattern whereby younger patients (18–25 years) and older (36 and above years) said “yes” they would like all the risk disclosed, while the middle age groups (26–35 years) said “no” they will not like the disclosure of all risks. This response comparison was statistically significant (*p* = 0.001). There was also a significant association with the level of education (*ρ* = 0.04), where patients with higher education levels reported that they would also like the disclosure of “all risks.”

### 3.5. General Knowledge of IC Regulations by Patients

When asked about the age of consent to treatment by children in South Africa, most participants (65.9%, *n* = 85) answered wrongly by choosing 18 years, some participants (10.9%, *n* = 14) selected 21 years, and two (1.6%) chose 15 years. Twenty-four participants (18.6%) reported that they “do not know.” Only three (2.3%) participants correctly answered 12 years (Table 5).

## 4. Discussion

This study explored patients' experience on practice and applicability of IC in African traditional Medicine (ATM) in Msunduzi and eThekwini municipalities, South Africa, in order to evaluate whether key elements of IC such as full information disclosure, capacity, understanding, and volition are considered or being applied during ATM practice. Patients in this study were generally informed about their diagnoses (58.9%), benefits of treatment (79.8%), and recommended treatment (79.8%). Fewer were informed about risks of treatment (36.4%), right of refusal (3.1%), and risk of refusing recommended treatment (0.8%). Nevertheless, all the patients were satisfied with the information given to them and did not feel coerced to accept treatment. THPs and patients' agreements/consent to treatment were obtained verbally in all cases. Most of the participants (76.7%) sought assistance in deciding, and 81.4% reported they would like to know all treatment risks and be involved in decision-making around their health care during traditional medical practice (TMP). There was association and statistical significance between information disclosed to patients and sociodemographic characteristics such as education, marital status, and occupation. This means that *married* patients are more likely to get more information on treatment when compared with *single* patients. The level of education was also associated with more information disclosure, that is, patients with secondary and tertiary education were more likely to get more information disclosed to them when compared with patients with primary education. Furthermore, occupational status was associated with information disclosure to patients, in that employed patients were more likely to get more information disclosed to them, when compared with patients who were unemployed or self-employed. This could be due the fact that such patients were more confident and self-assured and were able to ask questions and get more details about their treatment.

This study shows that patients' experience regarding the existing consent element in ATM practice in the eThekwini and Msunduzi municipalities of South Africa appeared adequate. This is because patients reported that the level of information disclosure during TMP encounters or procedures with all the participants was enough. However, only two of the information headings required by the National Health Act 2003 [22] were provided to most patients. These were diagnoses and benefits of treatment [4–6, 9, 23]. There appeared to be poor communication on risks of treatment, and this appears to be a consistent problem in TMP as reported by others [35, 40, 41]. As postulated by Chatfield et al. [35], over the last thirty years, several potential risks associated with the use of herbal medications have received growing attention. In particular, there are concerns that only a tiny fraction of used species in herbalism have been subjected to rigorous testing under controlled conditions, and clinical studies of herbal medicines vary greatly in quality and usefulness [11, 12, 35, 40, 41]. This has limited the ability to analyse the traditional medications and establish the possible risks of such medications on patients. Furthermore, very few of the participants in this study were given information on the right of refusal (3.1%) and the risk of refusing recommended treatment (0.8%). This could be as a result of the fact that there is no binding legal and ethical framework in ATM that necessitates the disclosure of risk. This implies that, even though the respondents reported that they were satisfied with the information provided, they did not have enough information to freely accept or reject the treatment procedure. Another study in eThekwini municipality public hospitals showed that, even in Western or orthodox medical practical, there is poor communication on patients' right of refusal and risk of refusing recommended treatment [4, 6, 9]. In addition, another study among physiotherapists and their assistants by Aderibigbe and Chima practicing in eThekwini municipality in 2019 [42], indicated that there was better emphasis on disclosure of the benefits, rather than the risks of treatment, which was aimed at encouraging patients to accept rather than reject recommended treatments [42]. A similar scenario may be responsible for the less disclosure of risks by THPs to their patients as observed in this study. Regarding the understanding of information disclosed, all the participants reported that they understood the information provided by the THP and over 90% said they were able to ask questions about their treatment. This implies that the practice of IC in ATM in South Africa is generally consistent with the existing regulations on the practice of IC by health professionals [4, 6, 9, 22, 23]. Regarding voluntariness of consent, all the participants reported that they made the decision of their own free will, although most of them sought assistance from surrogates who were mostly family members and friends. This finding can relate with some of the studies done in Durban and other parts of Africa where it was reported that consent in African communities should take into account relational autonomy because communal decision-making may be more paramount when compared with individual autonomy [26, 30, 43]. Most patients reported the absence of coercion or undue influence in this study, with over 90% reporting that they were not afraid to say “no” if they felt uncomfortable with the recommended treatment. This is consistent with a study conducted in the same region with patients treated in KZN public hospitals [4, 6]. However, this observation is still unusual for a developing country in Africa, especially during TMP where the implementation of IC is not established and where cultural and family influences are thought to play a major role in healthcare decision-making generally [15], as reported from Nigeria [44, 45], Ghana [33], and Kenya [46]. This may indicate that South African patients who go to traditional healers are more aware of their rights, which may facilitate the wide application of the IC doctrine as stipulated in the National Health Act 2003 [22, 23], as well as the patients' rights charter displayed by law in all public hospitals in South Africa and widely available on the Internet [4, 47].

All participants in this study reported that there was no perverse influence on their decision-making such as financial inducement or threats. Most of the patients spent 20 to 30 minutes with THPs during therapeutic encounters, although it is not clear whether this time specifically was for the whole consultation or just specifically on explaining the treatment procedures and reaching an agreement. However, in studies from South Africa among patients seen at public hospitals, the majority of patients reported that they spent between 5 and 10 minutes during most clinical encounters including IC procedures [4, 6, 9, 23, 48].

Also, participants in this study showed poor knowledge of the current legal age for consent in South Africa as stipulated by current laws [23, 48], with only 2.3% of the participants identifying the current correct legal age of consent to routine treatment as 12 years [48]. This is similar to studies conducted with patients and even nurse practitioners at public hospitals in KZN, where there was also poor knowledge regarding the legal age of consent in South Africa [4, 6, 21, 23]. This could imply that, even when copies of the NHA (2003) are easily available on the Internet or in the public space in South Africa, most people are not keen on familiarizing themselves about local regulations or are not adequately educated about current and basic local laws. This should be improved by better public information of healthcare users in South Africa.

Overall, respondents in this study felt they received and understood adequate information during their encounter with traditional healers in the area of consent in TMP; however, most respondents said they wanted to be involved in decision-making around their treatment through informed decision-making. Furthermore, over 80% of the participants reported that they would like to know all the “material risks” associated with the treatment or procedure [4, 23]. This finding is consistent with studies conducted on the practice of IC in Western medical practice in the same region [4, 6] and other parts of African such as Nigeria [49] and Kenya [46] where patients felt that, while IC was important, they also wanted to be involved in decisions affecting their healthcare.

In an effort to regulate, promote, and develop or standardize the practice of African traditional medicine, some African countries are reviewing existing legal frameworks or putting in place new ones [50]. For instance, regulations were signed by the Minister of Health in 2015 to provide for training and registration of THPs with the Traditional Health Practitioners Council of South Africa (THPCSA) [19, 20, 51]. Furthermore, the South African parliament passed the Traditional Health Practitioners Act in 2007 [20]. South Africa also created the South African Medical Association task team in 2006 to promote collaboration between traditional healers and biomedical researchers. The ultimate aim of all this is to reduce the burden of disease and improve the quality of life for patients who go to traditional healers, which should also be the goal of any effective healthcare system [17, 51, 52]. Thus, it may be immoral to focus only on the availability and affordability of ATM to the vulnerable African populations [50, 51] that rely on THPs, ATM, and TMP without assessing its safety and to evaluate whether the benefits actually outweigh the risks and to see whether ATM and TMP will assist in reducing the disease burden within African communities [52] and assist in achieving the UN-SDGs [53], as well as achieving the WHO goal of universal health coverage [54], in Africa and other developing countries.

### 4.1. Statement of Limitations

Some potential limitations of this study were that it was conducted in a community around the metropolitan urban communities of South Africa (Durban and Pietermaritzburg) with a well-educated and knowledgeable population group by South African standards. Similar studies in a more rural environment or different cultural contexts may lead to different conclusions. Second, due to the population that was being studied, the possible sampling technique was purposive. With this, we used the information we had to recruit participants. This might impact on the findings of this study as we were not able to randomise the selection of participants. Furthermore, data from traditional healers (THPs) on the practice of IC would have further validated the information in the study. While such a study is currently ongoing and will be reported in another paper arising from this project, the unavailability of such data does not invalidate the findings from this aspect of the study as reported in this paper. Finally, the researchers minimized response bias by using neutral questions in the questionnaire and ensuring that data were collected and reported anonymously.

## 5. Conclusions

This study has shown that most patients who go to traditional health practitioners (THPs) in eThekwini and Msunduzi municipalities, KwaZulu-Natal province, South Africa, are generally aware of their rights to information disclosure and the need for IC as illustrated by this group of TMP users. Healthcare users who prefer THPs also want to be informed and participate in shared healthcare decision-making. This study also shows that traditional medicine can survive the formal application of the IC process since the study has demonstrated that most legal requirements of IC are already applied during ATM practice in South Africa. But there may be need for ATM and TMP to be standardised. Patients and THPs also need more education regarding basic local laws, such as the current age of consent to routine medical treatment and other aspects of human rights. Finally, there is a need to further educate patients and THPs on the legal requirement of IC before its implementation. This will assist in enhancing patient-THP relationships and protect individual rights and human dignity.

## Figures and Tables

**Table 1:** Sociodemographic characteristics of participants (*N* = 129).

Characteristics	Number (*n*)	Percentage
*Age group (in years)*		
18–25	13	10.1
26–35	50	38.8
36–45	38	29.5
46 and above	28	21.7

*Relationship to patient*		
Self	127	98.4
Parent	2	1.6
Guardian	0	0.0

*Gender*		
Male	49	38.0
Female	80	62.0

*Marital status*		
Married	38	29.5
Single	81	62.8
Divorced	8	6.2
Separated	2	1.6

*Language spoken*		
*English speakers*		
No	118	91.5
Yes	11	8.5

*IsiZulu speakers*		
No	0	0.0
Yes	129	100.0

*Level of education*		
Primary	4	3.1
Secondary	62	48.1
Tertiary	63	48.8

*Occupation*		
Unemployed	47	36.4
Self-employed	20	15.5
Employed	62	48.1

*Monthly earnings (Rand)*		
No earnings	38	29.5
Less than R1000	7	5.4
Between R1001 and 3000	9	7.0
Between R3001 and 5000	1	0.8
Between R5001 and 10,000	9	7.0
Over R10,000	13	10.1
Do not know/no disclosure	52	40.3

**Table 2:** Information disclosed to a patient by traditional healers.

	Yes		No	
	*N*	%	*N*	%
*Explanation of the treatment by the traditional healers*	129	100.0	0	0.0%
*Information provided*				
Information on diagnosis	76	58.9	53	41.1
Information on treatment options	19	14.7	110	85.3
Information on risk	47	36.4	82	63.6
Information on benefits	103	79.8	26	20.2
Information on recommended treatment	103	79.8	26	20.2
Information on right of refusal	4	3.1	125	96.9
Information on risk of refusing recommended treatment	1	0.8	128	99.2

*Probing questions*				
The language used to explain procedure or treatment IsiZulu	129	100.0%		
Was the treatment explained by the THP	129	100.0%	0	0.0%
What method was used to explain (words)	129	100.0%	0	0.0%
Was the information provided understandable	129	100.0%	0	0.0%
Did you ask questions concerning the treatment or procedure	126	97.7%	3	2.3%

**Table 3:** Association between IC scores and demographic factors.

	IC score				Total	*p* value
	Low (<3)		High (≥3)			
	*N*	%	*N*	%		
*Age of respondent* (in years)						
18–25	3	23.1	10	76.9	13	0.54
26–35	18	36.0	32	64.0	50	
36–45	11	28.9	27	71.1	38	
46 and above	6	21.4	22	78.6	28	

*Gender*						
Male	12	24.5	37	75.5	49	0.33
Female	26	32.5	54	67.5	80	

*Marital status*						
Single	31	38.3	50	61.7	81	0.004
Married/divorced/separated	7	14.6	41	85.4	48	

*Level of education*						
Primary	4	100.0	0	0.0	4	0.005
Secondary	14	22.6	48	77.4	62	
Tertiary	20	31.7	43	68.3	63	

*Occupation*						
Employed	11	17.7	51	82.3	62	0.02
Unemployed	18	38.3	29	61.7	47	
Self-employed	9	45.0	11	55.0	20	

**Note:** A *p* value of < 0.05 was considered statistically significant.

**Table 4:** Logistic regression on the association of IC scores and demographics.

	IC score				Total	Bivariate				Multivariable			
	Low (<3)		High (≥3)			*p* value	OR	95% CI		*p* value	OR	95% CI	
	*n*	%	*n*	%									
*Age of respondent* (in years)						** **			** **	** **			
18–25	3	23.1	10	76.9	13	Ref	Ref	Ref		Ref	Ref	Ref	
26–35	18	36.0	32	64.0	50	0.38	0.53	0.13	2.19	0.20	0.38	0.09	1.67
36–45	11	28.9	27	71.1	38	0.68	0.74	0.17	3.20	0.28	0.41	0.08	2.03
46 and above	6	21.4	22	78.6	28	0.91	1.10	0.23	5.31	0.76	0.77	0.15	4.02

*Gender*													
Male	12	24.5	37	75.5	49	Ref	Ref	Ref	Ref	Ref	Ref	Ref	
Female	26	32.5	54	67.5	80	0.33	0.67	0.30	1.50	0.63	0.80	0.33	1.96

*Marital status*													
Single	31	38.3	50	61.7	81		Ref	Ref	Ref	Ref	Ref	Ref	
Married/div/Sep.	7	14.6	41	85.4	48	0.004	3.63	1.45	9.10	0.02	3.24	1.20	8.74

*Level of education*													
Primary/secondary	18	27.3	48	72.7	66		Ref	Ref	Ref	Ref	Ref	Ref	
Tertiary	20	31.7	43	68.3	63	0.58	0.81	0.38	1.72	0.91	0.95	0.40	2.27

*Occupation*													
Unemployed	18	38.3	29	61.7	47	Ref	Ref	Ref	Ref	Ref	Ref	Ref	
Self-employed	9	45.0	11	55.0	20	0.61	0.76	0.26	2.19	0.61	0.74	0.24	2.34
Employed	11	17.7	51	82.3	62	0.02	2.88	1.20	6.92	0.06	2.49	0.98	6.36

Note: div/Sep. = divorced or separated; OR = odds ratio; CI = confidence interval.

**Table 5:** Patients' responses regarding IC regulations and process.

	Yes		No		DK		Total
	*n*	%	*n*	%	*n*	%	*n*
Time spent to explain the procedure or treatment							
5 to 10 min	40	31.0					
10 to 20 min	66	51.2					
20 to 30 min	23	17.8					
Would you like to know							
All of the risks	105	81.4					
None of the risks	21	16.3					
Some of the risks	1	0.8					
Do not know	2	1.6					
Were you advised that you could accept or reject treatment	118	91.5	8	6.2	3	2.3	129
Did you seek assistance in deciding to accept treatment	99	76.7	30	23.3	0	0.0	129
If yes, from whom you sought assistance							
Parent	29	22.5					
Husband	4	3.1					
Wife	11	8.5					
Family member	33	25.6					
Friend	22	17.1					
No assistance	30	23.3					
Did you make your choice of your own free will	129	100	0	0.0	0	0.0	129
Was enough information provided	129	100	0	0.0	0	0.0	129
Did you feel threatened or afraid to say no	6	4.7	122	94.6	1	0.8	129
Were you offered an incentive	0	0.0	129	100	0	0.0	129
How did you consent (verbally)	129	100	0	0.0	0	0.0	129
At what age can a minor consent							
12 years	3	2.3					
15 years	2	1.6					
18 years	85	65.9					
21 years	14	10.9					
Do not know	24	18.6					
If you do not like the treatment, can you change your mind	127	98.4	1		1		129

Note: DK means do not know.

## Data Availability

The data sets used/or analysed during the current study are available from the corresponding author on reasonable request.

## Supplementary Materials

Supplementary Materials Questionnaire for patients' perspective of African traditional medicine (ATM).
