# Supplementary material for: Patients' Experience on Practice and Applicability of Informed Consent in Traditional Medical Practice in KwaZulu-Natal Province, South Africa
Source: Evid Based Complement Alternat Med. 2022 Jan 20;2022:3674467. doi: 10.1155/2022/3674467 (PMC8794665; doi:10.1155/2022/3674467)
Supplement: Supplementary Materials — Questionnaire for patients' perspective of African traditional medicine (ATM). [file 3674467.f1.docx]

**APPENDICES**

**INTERVIEW SCHEDULE FOR PATIENTS**

**Questionnaire No. .**

**Questionnaire for Patients**

**1. Interviewer Initials: _______________________________**

**Date of Interview: ________________________________**

**Section A- Demographic characteristics**

2. Age of respondent: _________________________________

3. Relationship to patient:

( ) Self ( ) Parent

( ) Guardian ( ) Other, Please specify_______________

4. Gender

( ) Male ( ) Female

5. Marital Status

( ) Married ( ) Separated

( ) Single ( ) Widowed

( ) Divorced

6. Status of Minors:

( ) Orphaned ( ) Living with parents ( ) Living on my own

1. Languages spoken:

| A: South Africa (KZN) | B: Nigeria (Akwa Ibom) |
| --- | --- |
| English Y or N | English Y or N |
| IsiZulu Y or N | Ibibio Y or N |
| IsiXhosa Y or N | Annang Y or N |
| Afrikaans Y or N | Eket Y or N |
|  | Oron Y or N |

Other, Please specify _______________________

9. Level of Education Completed

( ) None

( ) Primary

( ) Secondary

( ) Tertiary

10. Occupation

( ) Unemployed

( ) Self employed

( ) Employed

( ) Other – please specify ______________________________

11. How much do you earn in a month:

|  | A: South Africa (KZN) | B: Nigeria (Akwa Ibom) |
| --- | --- | --- |
|  | ( ) No earnings | ( ) No earnings |
| ( ) Less than | ZAR 1000 | NGN 20,000 |
| ( ) Between | ZAR1001- 3000 | NGN 20,000 – 70,000 |
| ( ) Between | ZAR 3001- 5000 | NGN 75,000 – 120,000 |
| ( ) between | ZAR 5001- 10,000 | NGN 125,000 – 240,000 |
| ( ) Over | ZAR 10,000 | NGN 240,000 |
| ( ) Don’t know/Refuse to disclose |  |  |

**Section B – Questions on Consent to treatment**

.

12. Have you been to traditional healer in the last 5years?

( ) Yes ( ) No. ( ) I do not remember

13. What type of healer did you visit?

( ) Sangoma Y or N

( ) Inyanga Y or N

( ) Traditional Midwives Y or N

( ) Traditional Obstetrics Y or N

( ) Traditional Surgeon Y or N

13. Name of the area of Traditional medicine in which you are or were receiving treatment:

( ) Herbalism Y or N

( ) Therapeutic Dieting Y or N

( ) Hydrotherapy Y or N

( ) Bone-setting Y or N

( ) Massage Y or N

( ) Surgical Procedures Y or N

( ) Childbirth Y or N

13. Did the Traditional doctor explain the treatment/ medical procedure to you?

( ) Yes ( ) No ( ) I do not remember

14. Please tell us the information that was provided to you?

( ) Diagnosis Y or N ( ) Risks Y or N

( ) Treatment Options Y or N ( ) Benefits Y or N

( ) Recommended Treatment Y or N ( ) Right of refusal Y or N

( ) Risks of refusing recommended treatment Y or N

Any additional information (please specify) ________________________________

________________________________________________________________

1. How much time did the traditional doctor take to explain the procedure or treatment?

( ) < 5 minutes ( ) 5-10 minutes

( ) 10-20 minutes ( ) 20-30 minutes

( ) > 30 minutes ( ) None

1. In what language was information on the treatment or procedure provided?

| A: South Africa (KZN) | B: Nigeria (Akwa Ibom) |
| --- | --- |
| ( ) English | ( ) English |
| ( ) IsiZulu | ( ) Annang |
| ( ) IsiXhosa | ( ) Ibibio |
| ( ) Afrikaans | ( ) Eket |
|  | ( ) Oron |
| ( ) More than one language. Please specify__________ | ( ) More than one language. Please specify____________ |
| ( ) Other, specify____________ | ( ) Other, specify___________ |

1. Did the traditional doctor explain the treatment that he/she would provide?

( ) Yes ( ) No ( ) I do not remember

1. Which of the following methods did the traditional doctor use to explain the treatment? Please tick.

( ) Words ( ) Diagrams

( ) Pictures ( ) Interpreter ( ) None

1. Did you understand the information provided?

( ) Yes ( ) No ( ) I do not remember

1. Did you ask any questions concerning the treatment or procedure?

( ) Yes ( ) No ( ) I do not remember

If No, Why not? ______________________________________________________

1. If you had had a choice would you like to know all the risks or some of the risks involved in the treatment or procedure?

( ) Yes, I would like to know all the risks, ( ) No, I would not like to know the risks

( ) I would like to know only some of the risks ( ) Don’t know

1. Please explain your choice ____________________________________________

____________________________________________________________________

1. Were you advised that you could accept or reject the treatment or procedure?

( ) Yes ( ) No ( ) Don’t remember

1. Did you seek assistance in reaching a decision whether to accept or reject the treatment or procedure?

( ) Yes ( ) No ( ) Don’t remember

1. If yes, why did you seek assistance?

____________________________________________________________________

____________________________________________________________________

1. Please specify from whom you sought assistance from

( ) Parent ( ) Child

( ) Husband ( ) Wife

( ) Family member ( ) Friend

( ) Other, specify________________________________

1. Did you make your choice of your own free will?

( ) Yes ( ) No ( ) Don’t Remember

1. If **No**, Please explain

_______________________________________________________________________

1. Do you think the amount of information provided to you was enough to enable you to make an informed choice?

( ) Yes ( ) No ( ) Don’t know

1. Did you feel threatened or afraid to say no?

( ) Yes ( ) No ( ) Don’t Remember

If yes, please explain______________________________________________________

1. Were you offered an incentive or persuaded to accept any particular treatment?

( ) Yes ( ) No ( ) Don’t remember

If yes, please explain______________________________________________________

1. How did you give consent?

( ) Verbally ( ) Written ( ) Don’t remember

**Section C- Generic questions on informed consent**

1. Do you have any suggestions or recommendations regarding informed consent in traditional medicine?

________________________________________________________________________________________________________________________________________________________________________________________________________________________________________________________________________________________________

1. At what age can a minor (young person) consent to routine medical treatment in South Africa? (Please choose one)……

12 years ( ) 15 years ( ) 18 years ( ) 21 years ( ) Don’t know ( )

1. At what age can a minor (young person) consent to routine medical treatment in Nigeria? (Please choose one)……

12 years ( ) 15 years ( ) 18 years ( ) 21 years ( ) Don’t know ( )

1. Finally, if you do not like the treatment that a traditional medical doctor is providing to you or your child, or if you think it is not working for you. Can you change your mind or refuse the treatment at any time?

( ) Yes ( ) No ( ) Don’t know

If **No**, Please tell us why? ___________________________________________

________________________________________________________________
